# Recurrent Enlarging Mesenteric Desmoid Tumor following Remote Surgical Resection

**DOI:** 10.1155/2017/2312617

**Published:** 2017-12-18

**Authors:** Connie Hapgood, Allison DeLong

**Affiliations:** ^1^Department of Radiology, Memorial University, St. John's, NL, Canada; ^2^Faculty of Medicine, Memorial University, St. John's, NL, Canada

## Abstract

Intra-abdominal desmoid tumors are commonly associated with genetic syndromes such as familial polyposis coli. Radiological cross imaging studies such as CT and MRI are used in the preoperative work-up of such tumors. Postoperatively, CT and MRI are useful in the assessment of recurrent desmoid tumors. MRI is more accurate in tissue characterization. Where possible, surgical resection remains the standard first-line treatment. For patients where surgery is not possible, or the resection margins are not clear, other forms of treatment are possible. These include hormonal and nonhormonal options. We present a case of a recurrent sporadic intra-abdominal (mesenteric) desmoid tumor. Such an entity is rare with few cases reported in the literature. Treatment options regarding intra-abdominal desmoid tumors will be discussed.

## 1. Introduction

Desmoid tumors are deep-seated monoclonal myofibroblastic tumors that account for approximately 0.03% of all neoplasms [1–3]. Desmoid tumors are histologically benign and will not metastasize; however they can display an aggressive growth pattern. Desmoid tumors can be grouped as extra-abdominal, within the abdominal wall, or intra-abdominal, extra-abdominal being the most common [4]. Desmoid tumors can occur sporadically; however they are most commonly associated with familial adenomatous polyposis (FAP) syndrome [4]. Such predisposing factors as prior surgery or trauma and high estrogen states have also been associated with the development of desmoid tumors. Abdominal wall desmoids are often associated with scar tissue. Intra-abdominal desmoid tumors tend to be associated with a diagnosis of FAP. Sporadic desmoid tumors are rarely intra-abdominal, most commonly occurring in the extremities. Desmoid tumors tend to have a high rate of recurrence even with total local resection. Sporadic desmoid tumors tend to reoccur less frequently in comparison to desmoid tumors associated with FAP [5]. Clinical presentation varies depending on the size and location of the tumor. Most patients are asymptomatic. Intra-abdominal desmoid tumors can be associated with abdominal pain and bowel obstruction.

## 2. Case Report

**Figure 1:**
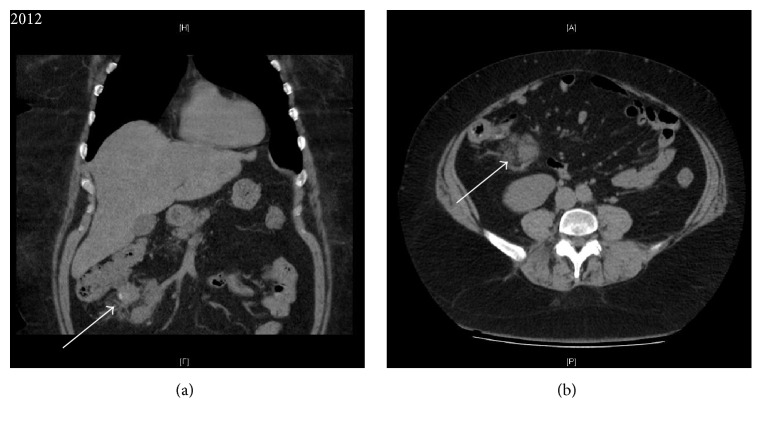
2012 Coronal enhanced (a) and axial enhanced (b) CT showing hypovascular solid mass (white arrow) in the mesentery of the right lower quadrant.

47-year-old female presented to our Emergency department in 2012. She complained of progressive intermittent abdominal pain. Clinical exam revealed bilateral pitting edema and an elevated jugular venous pressure, with no history of familial polyposis or colorectal cancer. Laboratory work-up was noncontributory. CT revealed an ill-defined hypoattenuating solid mass in the small bowel mesentery (Figure 1), with slight displacement of adjacent small bowel loops and mild enhancement.

The mass was resected surgically along with a right hemicolectomy and pathology reported it as a desmoid tumor. Mass was noted to have clear margins on pathology. No additional treatment was given. The patients immediate postoperative course was uneventful.

**Figure 2:**
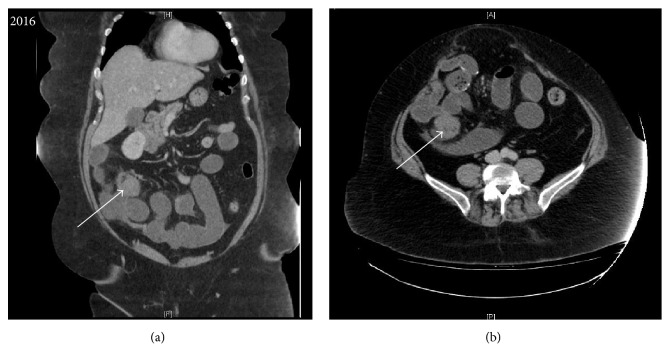
Coronal enhanced (a) and axial enhanced (b) CT performed 4 years later, showing recurrent hypovascular solid mass (white arrow) in the mesentery of the right lower quadrant.

Four years later, the patient returned to the Emergency department with a small bowel obstruction. There was no additional change in medical history. CT was repeated showing a recurrent irregular solid hypoattenuating mass in the mesentery of the right lower quadrant (Figure 2). The mass was located in close proximity to adjacent loops of ileum. The small bowel was distended with little gas in the colon consistent with a small bowel obstruction.

**Figure 3:**
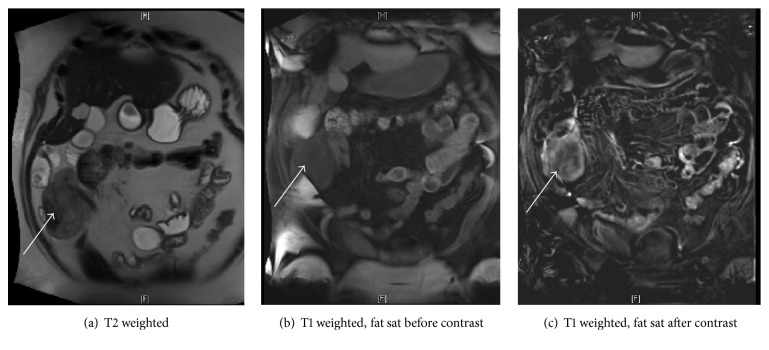
MRI T2 sequence (a), T1 unenhanced sequence (b), and T1 enhanced sequence (c), performed four months following most recent CT. This shows a solid mass (white arrow) of mixed signal intensity on T2, with mild enhancement, in the mesentery of the right lower quadrant that has increased in size from most recent CT.

MRI advised for further characterization of the right lower mesenteric mass. MRI (Figure 3) shows the mesenteric mass to be of heterogenous signal intensity on T2 weighted imaging, low in signal of T1 before contrast and slight enhancement after contrast. The findings suggest desmoid recurrence in the right lower quadrant.

In the four-month interval between most recent scans the mass had grown significantly from 5 × 4 cm on CT to 8 × 7 cm on MR (Figures 2 and 3, resp.). The patient underwent another laparotomy for definitive diagnosis. On surgical resection, the margins of the mass were noted to push against the edge of the serosal peritoneum. The mass was adherent to small bowel and involved 25–30 cm of the small bowel mesentery but was mobile.

**Figure 4:**
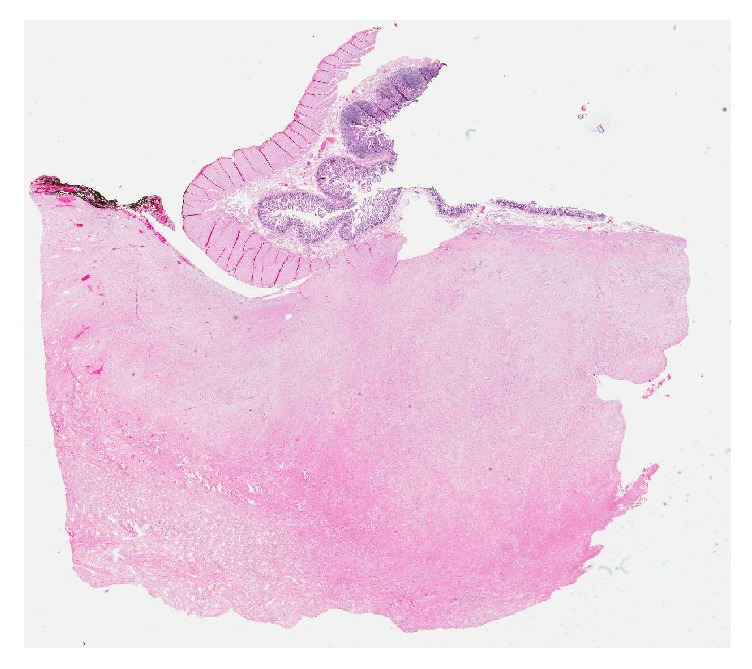
Low power view of the lesion in relation to the mucosa and muscular layer of the small bowel. The pink area at the bottom half of the image is all tumor.

**Figure 5:**
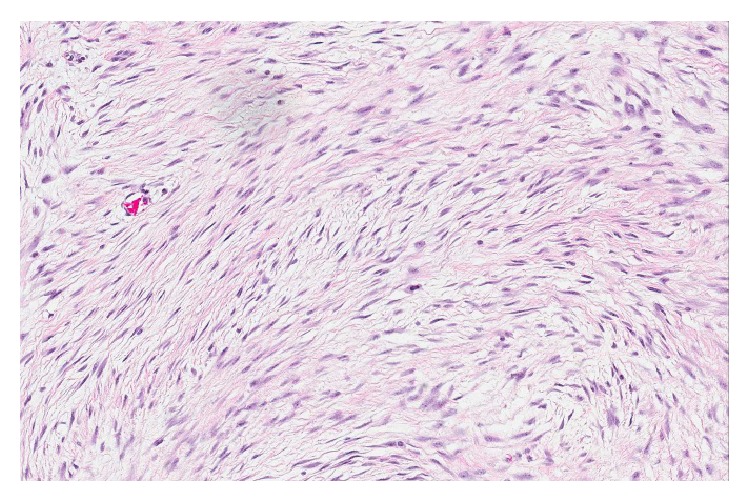
Medium power view (×20) is H&E stain of the tumor showing spindle cells (fibroblasts in sweeping fascicles).

**Figure 6:**
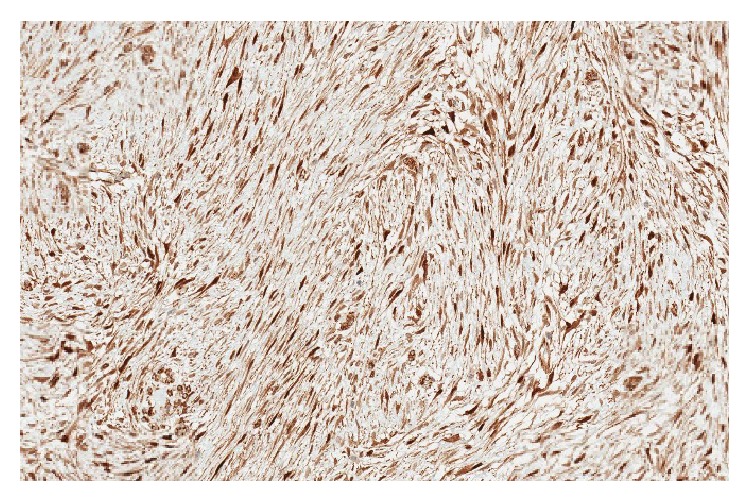
IHC stain for beta-catenin (×20) showing characteristic nuclear staining, confirming the diagnosis of fibromatosis.

On gross pathology, the mass was noted to be adherent to small bowel and firm. The mass was circumscribed and measured 9.2 × 7.3 × 7.2 cm. Focal areas of hemorrhage were noted near the periphery. The tumor did not invade surrounding fat or bowel (Figure 4). Microscopically the lesion was composed of fibroblasts arranged in sweeping fascicles (Figure 5). No atypia or pleomorphism was identified. Beta-catenin was diffusely positive (Figure 6).

Following surgical resection, no adjuvant therapy was administered. Follow-up with radiological imaging was planned.

## 3. Discussion

Desmoid tumors are exceedingly rare. Some identified risk factors include abdominal surgery/trauma, women of childbearing age (desmoid tumor growth is thought to be influenced by estrogen), and familial adenomatous polyposis/Gardner's Syndrome. Common presenting complaints are abdominal distention, pain, vomiting, and intestinal obstruction. However, most patients will present with a painless enlarging mass [3]. Intra-abdominal desmoid tumors are more commonly seen in patients with familial adenomatous polyposis. While sporadic desmoids more commonly present as an extra-abdominal mass [6, 7].

On CT, desmoid tumors commonly appear as a well-circumscribed homogenous lesion isodense or hyperdense relative to muscle [8]. The CT characteristics are not pathognomonic of desmoid tumors. Other mesenteric masses such as gastrointestinal stromal tumors (GIST) can have a similar appearance. The differential for such a mesenteric mass on CT would also include carcinoid, leiomyoma, leiomyosarcoma, and lymphoma. CT is useful preoperatively to assess involvement of adjacent organs and vasculature. Desmoids on MRI are of low signal intensity of T1 images and will often demonstrate heterogeneity on T2 images [9]. MRI is a useful tool in tissue characterization, commonly used in the cases of desmoid tumor recurrence. Histologically the tissue obtained from a desmoid biopsy would appear as intertwining bundles of spindle cells within a collagen matrix [10]. Sporadic desmoid tumors are commonly associated with somatic mutations of the beta-catenin gene while desmoids associated with familial adenomatous polyposis coli often contain mutations in the APC gene [11].

The course of mesenteric fibroblastic tumors/desmoid tumors is often unpredictable. They can be indolent and spontaneously regress while others can be very aggressive with rapid growth and mass effect [12]. They do not metastasize but may recur. Some studies quote recurrence rates as high as 40% [2]. Close observation is an acceptable strategy for stable asymptomatic patients. Complete surgical resection with wide surgical margins (at least 1 cm) is often the first line therapy for resectable tumors [7]. However even with complete surgical resection margins, desmoid tumors may recur. Furthermore, surgery may be difficult depending on the patient's comorbidities or location. For instance, resection of mesenteric desmoid tumors is often associated with resection of bowel and mesenteric vasculature increasing the risk of postop comorbidities. Most patients require an individualized treatment approach. In our patient, no adjuvant therapy was offered following surgical resection. Margins were free of tumor. In patients where the desmoid tumor is infiltrative with vital structures involved, or margins are not free of tumor, additional therapy may be warranted. There are hormonal and nonhormonal options for medical management. Tamoxifen is often used in combination with NSAIDs (indomethacin or sulindac) [11, 12]. Tamoxifen is a selective ER modulator that acts by binding to ER receptors and inhibiting cell proliferation. Alternatively, the analog Raloxifene can be used with prior studies showing positive treatment results [13]. NSAIDS can have partial or complete response. Chemotherapy is reserved for people who have aggressive nonsurgical tumors or in patients who are highly symptomatic when other therapy regimes are not feasible or have failed. Chemotherapy is typically given in doses individualized to the patient based upon a team approach. Furthermore, in patients who are not a candidate for surgery, tyrosine kinase inhibitor may be an option for treatment. Radiation treatment is an option, reserved mainly for extra-abdominal tumors due to the risk of radiation induced injury to intra-abdominal structures, for example, radiation induced enteritis.

## 4. Conclusion

Sporadic intra-abdominal tumors occur much less frequently when compared to intra-abdominal desmoid tumors associated with familial polyposis coli. Most sporadic tumors are extra-abdominal in location. Management of intra-abdominal desmoid tumors involves a multidisciplinary team approach. Asymptomatic patients are commonly closely observed for disease progression. Resection remains the first line of treatment. Hormonal and nonhormonal treatments remain an option in patients where resection is not feasible or in those where disease free margins are not achieved. Disease free survival following resection depends on tumor size (>5 cm), extra-abdominal location, margin resection status, and history of recurrence [6]. The recommendation for surveillance is imaging every 3–6 months for the first 2-3 years, then annually [2]. Sporadic desmoid fibromatosis of the mesentery is a rare occurrence. In our case, the recurrent tumor size was 9 cm, the excision margins were clear, and an immunohistochemical analysis of beta-catenin was positive. No additional adjuvant treatment initiated at this time; the patient is on long term clinical and radiological imaging surveillance.
